# One life ends, another begins: Management of a brain-dead pregnant mother-A systematic review-

**DOI:** 10.1186/1741-7015-8-74

**Published:** 2010-11-18

**Authors:** Majid Esmaeilzadeh, Christine Dictus, Elham Kayvanpour, Farbod Sedaghat-Hamedani, Michael Eichbaum, Stefan Hofer, Guido Engelmann, Hamidreza Fonouni, Mohammad Golriz, Jan Schmidt, Andreas Unterberg, Arianeb Mehrabi, Rezvan Ahmadi

**Affiliations:** 1 Department of General, Visceral and Transplantation Surgery, University of Heidelberg, Heidelberg, Germany; 2 Department of Neurosurgery, University of Heidelberg, Heidelberg, Germany; 3 Departments of Obstetrics and Gynecology, University of Heidelberg, Heidelberg, Germany; 4 Department of Anesthesiology, University of Heidelberg, Heidelberg, Germany; 5 Department of Pediatrics, University of Heidelberg, Heidelberg, Germany

## Abstract

**Background:**

An accident or a catastrophic disease may occasionally lead to brain death (BD) during pregnancy. Management of brain-dead pregnant patients needs to follow special strategies to support the mother in a way that she can deliver a viable and healthy child and, whenever possible, also be an organ donor. This review discusses the management of brain-dead mothers and gives an overview of recommendations concerning the organ supporting therapy.

**Methods:**

To obtain information on brain-dead pregnant women, we performed a systematic review of Medline, EMBASE and the Cochrane Central Register of Controlled Trials (CENTRAL). The collected data included the age of the mother, the cause of brain death, maternal medical complications, gestational age at BD, duration of extended life support, gestational age at delivery, indication of delivery, neonatal outcome, organ donation of the mothers and patient and graft outcome.

**Results:**

In our search of the literature, we found 30 cases reported between1982 and 2010. A nontraumatic brain injury was the cause of BD in 26 of 30 mothers. The maternal mean age at the time of BD was 26.5 years. The mean gestational age at the time of BD and the mean gestational age at delivery were 22 and 29.5 weeks, respectively. Twelve viable infants were born and survived the neonatal period.

**Conclusion:**

The management of a brain-dead pregnant woman requires a multidisciplinary team which should follow available standards, guidelines and recommendations both for a nontraumatic therapy of the fetus and for an organ-preserving treatment of the potential donor.

## Background

Brain death (BD) as "*coma dépassé*" was first defined by Mollaret and Goulon in 1959 [1], and it remains the medically and legally accepted framework for the diagnosis of death. Death is pronounced on the basis of well-defined clinical examinations followed by confirmatory technical tests. Recent improvements in life support technology and critical care management make it possible to maintain the patient's vital functions after BD. The question whether to offer life support to brain-dead patients and how long it should be provided has become a controversial ethical issue. The issue is still more complex when BD occurs during pregnancy. Of course, the incidence of BD in pregnant women is very low and there are only few case reports. As shown by Suddaby *et al*. [2], of 252 brain-dead patients, only 5 (2.8%) cases involved pregnant women between 15 and 45 years of age.

When confronted with BD in a pregnant woman, physicians must primarily focus on saving the life of the fetus, and therefore the treatment protocol should give special recommendations on how to support the mother in a way that she can deliver a viable and healthy child. After delivery, brain-dead pregnant women may also be candidates for organ donation. Therefore, two aspects must be considered in case of maternal BD: supporting the fetus until successful delivery and, if possible, supporting the brain-dead mother as an organ donor. Hence, if the mother and the fetus are regarded as two distinct organisms, maintaining the vital functions of a brain-dead pregnant patient may be ethically justifiable to support both the birth of a child and possible organ donation. In such a situation, various clinical disciplines such as neurosurgery, intensive care medicine, obstetrics, neonatology, anesthesiology, transplantation surgery and an ethics committee should work together to minimize maternal and fetal morbidity as well as mortality.

Since only a few reported cases are to be found in the medical literature, most approaches to managing a brain-dead mother remain experiential and relatively little publicized. In this article, we review the available cases of prolonged somatic support in brain-dead pregnant women and analyze when and under which circumstances the pregnancy should be maintained and what challenges are to be faced. To present a protocol to support such patients, we discuss the management of the brain-dead mother and fetus, related recommendations and legal and ethical issues.

## Methods

### Search strategy

We performed a systematic review of Medline (1975-2010), EMBASE (1982-2010) and the Cochrane Central Register of Controlled Trials (CENTRAL) (The Cochrane Library Issue 1, 2010) for relevant citations. Key words used in electronic searching included "maternal brain death," "pregnancy," "brain death," "management of brain death" and "fetal monitoring." Reference lists of retrieved relevant articles were screened for other studies. Additionally, to include all existent studies, we also contacted experts in the related fields of brain death and pregnancy.

### Study selection and data extraction

All studies which reported at least one case of maternal brain death during pregnancy were eligible for inclusion. We excluded studies dealing with pregnancy in a persistent vegetative state because brain death adds complexities to pregnancy that are very different from a persistent vegetative state, calling for different medical management and obstetric strategies, as well as other legal and ethical considerations. Furthermore, we excluded studies which only discussed ethical and legal issues and studies providing insufficient data. There were no language restrictions. One reviewer (ME) screened all titles and abstracts to assess whether they were potentially eligible for inclusion and whether full text was required. Then abstracts and full texts for all potentially eligible studies were reviewed by two researchers (ME and EK), who independently evaluated these articles and extracted their data. Any disagreement during study selection and the data extraction process was resolved by discussion with a third author (AM). According to our search of the medical literature, 30 cases of maternal BD (19 case reports and 1 case series) were reported between 1982 and 2010. The collected data included the age of the mother, the cause of BD, maternal medical complications, gestational age at BD, duration of life support and gestational age at delivery. In addition, we evaluated the indication of delivery, mode of delivery, neonatal outcome and organ donation by the mothers, as well as the transplant outcome. In our analysis, we particularly focused on the critical care management of brain-dead mothers such as respiratory and cardiovascular support, endocrinology and thermoregulation, nutritional support and organ donation, as well as aspects of obstetric management, including fetal monitoring.

### Statistical analysis

The statistical analysis was performed using SPSS 14.0 software for Windows (Stata Corp., College Station, TX, USA). All statistical data regarding maternal age, duration of maternal support, gestational age and fetal birthweight were expressed as means.

## Results

**Figure 1:**
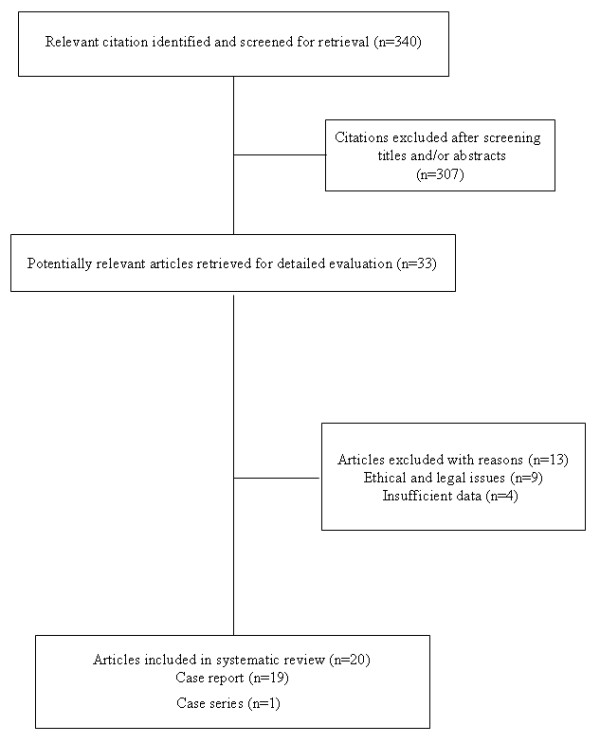
**Flow chart of abstracts and articles identified and evaluated during the review process**.

Figure 1 summarizes the process of literature identification and selection. According to our search, between 1982 and 2010, 19 case reports and 1 case series were published. In Table 1, we summarize the 30 reported cases of extended maternal life support after BD with pregnancy and the resulting neonatal outcomes.

**Table 1:** An overview of the reported cases of extended maternal somatic support after brain death (BD) including neonatal outcomes*

Study	Year/ country	Age of mother (yr)	Cause of BD	Gestational age at BD (wk)	Duration of life support (days)	Maternal medical complications	Indication for delivery	Gestational age at delivery (wk)	Mode of delivery	Neonatal outcome	Organ procurement	Transplant outcome
Dillon *et al*. [48]	1982/ USA	24	Meningitis	23	24	Thermovariability, DI	Fetal distress	26	C/S	Female, 930 gr, Apgar 8/8, IRDS	No	-
Heikkinen *et al*. [49]	1985/ Finland	31	ICH SAH	21	71	Thermovariability, pneumonia, hypotension, DI, bacteremia, panhypopituitarism	Maternal blood pressure fluctuation	31	C/S	Male, 1600 gr, Apgar 6/7, IRDS Normal at 8 mo.	No	-
Field *et al*. [14]	1988/ USA	27	CNS mass	22	63	Thermovariability, hypotension, panhypopituitarism, DI, ARDS, UTI, bacteremia	Septicemia, Growth retardation	31	C/S	Male, 1440 gr Apgar 8/8, IRDS, normal at 18 mo.	No	-
Bernstein *et al*. [33]	1989/ USA	30	Traumatic brain injury	15	107	Thermovariability, panhypopituitarism, pneumonia, DI	Suspicious for fetal distress	32	C/S	Male, 1555 gr Apgar 6/9, Mild hyperbilirubinemia, Normal at 11 mo.	No	-
Antonini *et al*. [50]	1992/ Italy	25	ICH	15	49	Panhypopituitarism, Pneumonia UTI, Hemodynamic instability Hyperglycemia anemia	Maternal death due to progressive hypotension	Not applicable	Not applicable	Intrauterine death	No	-
Nettina *et al*. [51]	1993/ USA	31	ICH	27	44	hypothermia; hypotension; decubitus ulcer, DI, pneumonia	Maternal hypotension	33	C/S	Male, 2083 gr Apgar 9	Yes	N.A.
Anstotz [52]	1993/ Germany	18	Accident	13	38	Severe infection	Not applicable	Not applicable	Not applicable	Spontaneous abortion at 19 weeks (autopsy refused)	No	-
Beguin [53]	1993/ Switzerland	20	ICH	20	3	No complication	Not applicable	Not applicable	Not applicable	Intrauterine death	Yes	N.A.
Wuermeling [54]	1994/ Germany	18	Traffic accident	14	N.A	infection	N.A.	N.A.	N.A.	Intrauterine death	N.A.	-
Iriye *et al*. [55]	1995/ USA	35	ICH after cocaine	30	2	hypotension	Maternal blood pressure fluctuation	30	C/S	Male, 1610gr, Apgar 7/8	No	-
Vives *et al*. [56]	1995/ Spain	25	Meningitis	27	1,5	Hypotension, sepsis, DIC, cardiac arrhythmia	Maternal hypotension	27	C/S	Male, 1150 gr, Apgars 7/10, IRDS, normal at 14 mo.	No	-
Catanzarite *et al*. [32]	1997/ USA	25	ICH	25	25	Hypotension, ARDS, DI, panhypopituitarism, pneumonia,	Fetal distress	28	C/S	Male, 1315 gr Apgar 3/7 fungemia	No	-
Lewis *et al*. [42]	1997/ USA	20	SAH	25	54	Hypotension, DI bacteremia,	Sufficient fetal lung maturity	32	C/S	Not available	Yes	No complication after 1 year
Suddaby *et al*. [2]	1998/ USA	Range from 15 to 45 (11 cases)	5 cases: ICH 1 case: Hematoma 1 case: Aneurysm 1 case: Amniotic embolus 1 case: Glioblastoma 1 case: Cardiac arrest 1 case: Gunshot	Range from 2 to 40	N.A	Hypotension DI Anemia Hypernatremia Hyperglycemia Hypocalcemia hyperchloremia	N.A	N.A	N.A	N.A	In five mothers	Of 25 donated organs (5 heart, 5 liver, 10 kidney, 5 pancreas), only one liver and one pancreas graft lost.
Spike [57]	1999/ USA	20	ICH	16	100	Panhypopituitarism, DI, Thermovariability Hypotension	Unusual pattern of the placenta in ultrasound	31	C/S	Male, 1440gr Apgar 8/8	No	-
Beca *et al*. [58]	1999/ Chile	26	ICH	17	5	Hemodynamic instability Fiber	Maternal death due to resistance hypotension	Not applicable	Not applicable	Intrauterine death	No	-
Lane *et al*. [59]	2004/ Ireland	26	Cerebral venous sinus thrombosis	13	8	DI, pneumonia, Hyper- and hyponatraemia	Not applicable	Not applicable	Not applicable	Intrauterine death at 14 weeks	Yes	N.A.
Hussein *et al*. [60]	2006/ UK	33	ICH	26	14	Hypertension, bradycardia, Chest infection, Hyperglycemia, serum cortisol reduced	Progressive oligohydraminos	28	C/S	Male, 1285 gr, breathing difficulties Normal at 24 mo.	No	-
Souza *et al*. [61]	2006/ Brazil	40	ICH	25	25	Panhypopituitarism, hyperglycemia DI, hypotension, bradycardia, hypothermia, pneumonia,	Progressive oligohydraminos, brain sparring	29	C/S	Male, 815 gr Apgars:9/10 Normal at 3 mo.	Yes	N.A.
Mejia *et al*. [62]	2008/ Argentina	29	ICH	17	56	DI Panhypopituitarism, Pneumonia UTI, Hemodynamic instability	Maternal hypotension & Cardiac arrest	25	C/S	450 gr Premature Birth complication, Candida infection Died at day 30	No	-

*ICH, intracranial hemorrhage; SAH, subarachnoid hemorrhage; DI, diabetes insipidus; ARDS, acute respiratory distress syndrome; UTI, urinary tract infection; C/S, cesarean section; IRDS, infant respiratory distress syndrome; N.A: not available.

### Maternal and obstetric outcome

In the 30 reported cases, the maternal mean age at the time of BD was 26.5 years. Only three mothers were in the high-risk pregnancy category (age <18 or age >35 yr) with respect to their age. Two mothers were 18 years old and a third one was 40 years old at the time of pregnancy. Trauma was the cause of BD in 4 of 30 mothers, and the other 26 died of nontraumatic brain injuries. The mean duration of maternal support was 38.3 days (range, 2-107 days). In two cases, children were delivered on the second day after BD was diagnosed. Conversely, in two reports, mothers were supported for more than 100 days before delivery. The mean gestational age at the time of BD was 22 weeks (range, 1-40 wk). In 10 of 19 reported cases, the baby was delivered later than week 28. The mean gestational age at delivery was 29.5 weeks (range, 26-33 wk). During extended life support, patients developed several complications, including infection, hemodynamic instability, diabetes insipidus (DI), panhypopituitarism, poikilothermia, metabolic instability, acute respiratory distress syndrome and disseminated intravascular coagulation (Table 1). The indications for delivery in all reported cases were maternal or fetal difficulties, including maternal hemodynamic instability (seven cases), fetal distress (three cases), oligohydramnion (two cases), intrauterine growth retardation (one case) and abnormal pattern of the placental structure (one case). In two cases in which maternal BD began at week 13 of gestational age, spontaneous abortion occurred at weeks 13 and 19. In four cases, there was intrauterine death. A cesarean section was the mode of delivery in all cases which resulted in live-born fetuses (Table 1).

### Fetal and neonatal outcome

In 12 (63%) of 19 reported cases, the prolonged somatic support led to the delivery of a viable child. We did not find any information about the fate of the fetuses in the published case series. Children who were born included 1 female and 10 male infants. No information regarding sex was given about one infant. The average birthweight was 1,384 g (range, 815-2,083 g), and the mean Apgar score was 7 and 8 at 1 and 5 minutes, respectively. Congenital defects were reported for only one infant, who was diagnosed with fetal hydantoin syndrome resulting from previous chronic phenytoin usage by the mother. Four infants required temporary mechanical ventilation because of neonatal respiratory distress syndrome or pneumonia. Fungemia was diagnosed in one infant, and he was treated with amphotericin B. However, not every infant was sufficiently followed to determine the long-term effects of prolonged maternal life support. Postnatal follow-up up to 24 months was available only for six infants. All of them developed normally and apparently had no problems related to their exceptional intrauterine circumstances (Table 1).

### Organ procurement and transplant outcome

In three reported cases after successful delivery, organ donation from the brain-dead mother was carried out. In two cases, organ procurement was accomplished after the intrauterine death of the fetus. In yet another five cases, organ donation was performed, but no report about the status of the fetus was provided. In six patients, consent was given by the patient's family to donate heart, lung, liver, pancreas and kidneys. In four donors, no information was given concerning donated organs. The 1-year graft survival in the reported cases was excellent. Only one liver and one pancreas were lost in two patients owing to their primary nonfunction. Finally, in all cases, maternal somatic support was ended either after delivery or after organ donation (Table 1).

## Discussion

**Figure 2:**
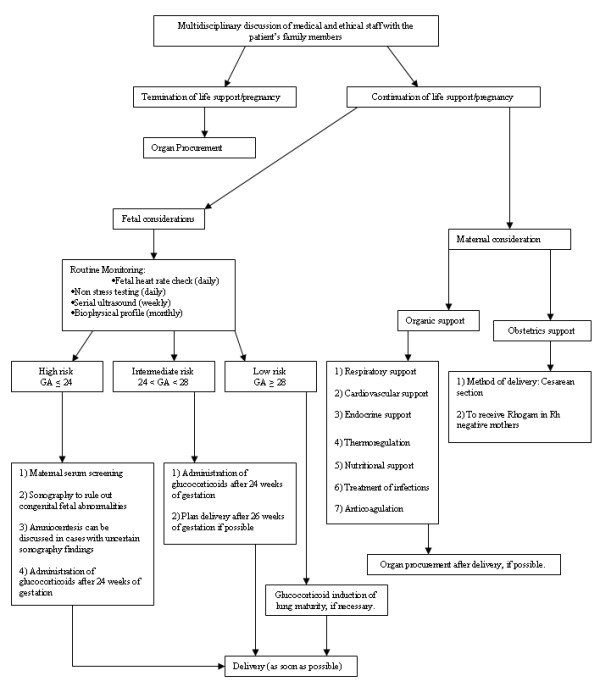
**Recommendations for the management of maternal brain death**. GA, gestational age.

Clinically, following the onset of BD, it is possible to sustain a brain-dead mother's somatic functions over a longer period. Manifold physiological changes occurring during pregnancy and brain death, as well as the prolonged hospital stay after BD, present enormous challenges, however, both for the treating clinicians and for the family. The important question is from which gestational age onward should the pregnancy be supported? At present, it seems that there is no clear lower limit to the gestational age which would restrict the physician's efforts to support the brain-dead mother and her fetus. As reported by *Slattery et al*. [3], a fetus born before 24 weeks of gestation has a limited chance of survival. At 24, 28 and 32 weeks, a fetus has approximately a 20-30%, 80% and 98% likelihood of survival with a 40%, 10% and less than 2% chance of suffering from a severe handicap, respectively. Therefore, depending on maternal stability and fetal growth, the decision must be made on an individual basis. According to our findings, prolonged somatic support can lead to the delivery of a viable child with satisfactory Apgar score and birthweight. Such children can also develop normally without any problems resulting from their intrauterine conditions. Furthermore, after the delivery, mothers could be considered as potential organ donors. In Figure 2, we summarize the recommendations for the critical care management of brain-dead pregnant women. This schema is not a definitive guideline, because the technical support and the experience of the responsible medical team must also be taken under consideration. Also, the number of reported cases is too small to define the rate at which intensive care support of the brain-dead mother can result in a healthy infant. The percentage of successful cases cannot be determined, because there are no reports describing failure of intensive maternal support from all medical centers. Finally, it cannot be established whether a relative infrequency of cases such as those that we found in the published literature reflects the rarity of the event, perfect success in all prior situations, reluctance to initiate intensive efforts required to support the brain-dead patient or simply publication bias.

However, we maintain that the management of a brain-dead pregnant woman should follow the existent standards, guidelines and recommendations both for nontraumatic therapy for the fetus and organ-preserving treatment for the donor [4-6]. What follows here is the summary of these guidelines and recommendations.

### Cardiovascular support

In the initial phase of BD, tachycardia was detected in less than half of the patients [7,8]. However, subsequently the heart rate slowed in all of these patients as factors such as hypothermia and subclinical myocardial hypoxia antagonized the sympathetic activation occurring during the initial phase of BD [7]. Hypertension in this situation is a rare, usually self-limiting event. In prolonged hypertension, short-acting substances such as urapidil or nitroprusside were applied [9,10]. Typically, at some point, BD patients also develop hypotension [9]. The initial treatment for hypotension consists of aggressive fluid replacement, which is usually done with crystalloids such as lactated Ringer's solution in normal (0.9%) or half-normal (0.45%) saline solutions. Recent studies suggest that to keep intravascular volume and colloid oncotic pressure within physiological ranges [9], hydroxyethyl starch can also be applied in case of a negative effect on the renal graft function [11]. However, it must be kept in mind that low oncotic pressure and hypoalbuminemia can cause pulmonary edema [12,13]. Field *et al*. [14] recommended that in case of pulmonary edema a Swan-Ganz catheter be used to differentiate cardiogenic pulmonary edema from acute respiratory distress syndrome (ARDS) and to guide fluid management. In addition, extensive hemodynamic monitoring such as Pulscontour Continuous Cardiac Output (PiCCO™) should be considered [4,15]. Fluid-resistant hypotension can be treated using continuous intravenous dopamine receptor agonists, which should be titrated until a mean arterial pressure of 80 to 110 mmHg is reached [16].

### Respiratory support

In maternal BD, special attention needs to be paid to mechanical ventilation. To facilitate the elimination of carbon dioxide from the fetus and as a result of the progesterone effect on the respiratory center, the pregnant mother develops hypocarbia mediated by an increase in tidal volume and respiratory rate. Hypocarbia is compensated by an increase in excretion of bicarbonates by the kidneys [17]. Maternal carbon dioxide tensions, a tidal volume and respiratory rate should be maintained in the normal pregnancy range of 28 to 31 mmHg, 6 to 8 mL/kg and 10 to 12/min, respectively [18]. The fraction of inspired oxygen should be kept in a range maintaining the arterial oxygen saturation above 90%.

### Endocrine support

Seventy-eight percent of brain-dead patients who were kept alive for more than a few days developed central diabetes insipidus (DI) resulting from posterior pituitary gland failure [19]. Administration of vasopressin and aggressive volume replacement should be performed for the treatment of DI [4]. Howlett *et al*. [20] reported a decrease in serum triiodothyronine (T3) in 81% and in serum thyroxin (T4) in 29% of BD dead organ donors. Therefore, especially in brain-dead pregnant women T3/T4 substitution should be adjusted according to laboratory examinations. Adrenal insufficiency causes hypotension and should be treated with methylprednisolone. To avoid prolonged exposure of the fetus to glucocorticoids during maternal somatic support, prednisone or methylprednisone should be used, as they do not readily cross the placenta [21]. Furthermore, since hyperglycemia is also observed during BD as a result of stress-related peripheral insulin resistance, insulin substitution may be needed to achieve normoglycemia [12].

### Thermoregulation

According to Smith *et al*. [22], the majority of brain-dead patients develop hypothermia. It is recommended that the patient be rewarmed passively using warming blankets or by warming of fluids. Following infections, brain-dead patients might also develop hyperthermia. In general, the inability to maintain body temperature and poikilothermia (body temperature that is dependent on the environment's temperature) accompany brain-dead patients [23,24].

### Nutritional support

The nutritional needs of a pregnant woman before and after BD are not the same. Basal energy expenditure (BEE) in pregnancy is 655 Kcal + (9.6 × weight (kg) + [1.8 × height (cm) -- 4.7 age (year)]) [25-27]. A weight gain of 10 to 15 kg accompanies a normal pregnancy [12]. A brain-dead pregnant woman will expend about 75% of a healthy pregnant woman's BEE [27]. Nutritional support should be calculated by maternal serum alimentary values, the weight of the mother and the growth of the fetus. Owing to reduced motility of the gastrointestinal tract in brain-dead patients, special attention should be paid to the management of gastric reflux. Total parenteral nutrition (TPN) during BD in pregnant mothers needs to support a positive nitrogen balance, maternal weight gain, and normal fetal growth and birthweight [28]. The recommended daily allowance for protein during pregnancy is 0.8 g kg^-1 ^day^-1 ^(the normal intake for an average healthy adult) plus an additional 1.3, 6.1 or 10.7 g kg^-1 ^day^-1 ^for the first, second or third trimesters, respectively [29,30]. In addition, 20-25% of nonprotein calories should be from fat [29].

### Infection

There are three sources of infection which should be taken into account during prolonged somatic support: ventilators causing recurrent pneumonia, urinary catheters resulting in bladder and kidney infections and intravascular catheters as a source of septicemia [31]. The majority of reported organisms were typical of nosocomial intensive care unit infections such as *Staphylococcus aureus*, *Actinobacter*, *Pseudomonas*, *Hemophilus influenza *and fungal pathogens [32,33]. These infections are usually resistant to antibiotics, and their treatment is challenging. Maternal infections must be treated aggressively with the most effective substances, rather than opting for using substances safe for the fetus, which in turn may not effectively treat the infection [34-36].

### Prophylactic anticoagulation

The risk of developing deep vein thrombosis is greater during pregnancy because of immobility and flaccid paralysis following BD. Recommended is prophylactic anticoagulation as it is efficacious for the mother and safe for the fetus. For venous thromboembolic disease treatment or prophylaxis during pregnancy, low molecular weight heparin appears to be as safe and effective as unfractionated heparin [37].

### Obstetric considerations

In maternal BD, it is recommended to screen the mother's serum and to examine carefully the fetus by ultrasound to establish that there are no malformations or pathologic findings in the fetal development and no chromosomal abnormalities. In cases with uncertain findings, amniocentesis should be discussed with family members, since the results of these screenings may influence their decisions [17]. In addition, laboratory tests including complete blood cell count, electrolytes such as Na^+^, Ca^2+^, K^+^, creatinine, urea, liver enzymes, retinol-binding proteins, albumin, prealbumin, transferrin and urine analysis should be periodically performed. After 24 weeks of gestation, glucocorticoids should be administered for fetal lung maturation and prophylaxis of fetal respiratory distress syndrome [38,39]. To prevent preterm uterine contractions, in particular in the early weeks of gestation when no fetal lung maturation is yet provided, tocolytic interventions may be needed. Calcium channel blockers and prostaglandin inhibitors are effective and well tolerated and are therefore preferred to β-mimetic agents [37,40]. A prolongation of the pregnancy should continue until at least 26 weeks of gestation with a possible second application of glucocorticoids. If maternal and fetal status remain stable, further prolongation of the pregnancy until at least 28 weeks of gestation should be attempted. According to the reported literature, after 32 weeks of gestation and under glucocorticoid-induced fetal lung maturity, no further prolongation of a pregnancy seems necessary. The optimal method of delivery in prolonged maternal somatic support is by cesarean section, as it ensures the least traumatic birth for the fetus. The optimal timing for a cesarean section can be estimated by amniocentesis assessing fetal lung maturity [41].

### Fetal and neonatal considerations

The gestational age and the condition of the fetus, above all lung maturity, are the two most important factors affecting fetal outcome. The majority of studies reported routine and complex fetal monitoring such as daily fetal heart rate monitoring using cardiotocography and nonstress testing. Serial ultrasound examinations to evaluate the fetoplacental unit, including biometric estimations as well as morphologic studies on the placental structure and the amnion fluid, should be performed weekly to assess intrauterine fetal growth [39,42-44].

### Organ donation and transplant outcome

After the delivery of the fetus, a brain-dead mother should be considered as a potential organ donor. Multiorgan instabilities and extensive critical care therapy lasting for weeks may have endangered the organs and caused complications in the recipients. Nevertheless, if one or more organs are still functioning at the time of delivery, the feasibility of organ donation in such catastrophic cases should not be ignored. As reported by Suddaby *et al*. [2] in a retrospective review of 252 brain-dead potential donors from 1990 to 1996, five of seven pregnant women functioned as organ donors for 20 transplant recipients. For all of those patients, excellent patient and graft outcomes were reported.

### Ethical and legal issues

Many ethical and legal questions arise in cases of maternal BD. Although it was not the focus of this review, we briefly discussed various aspects of ethical and legal issues such as "the mother's body as a cadaveric incubator," "mother as the organ donor and fetus as the recipient" and the concern for "possible damages to the fetus" [31,45]. Some professionals believe that it is not ethically acceptable to maintain the mother's body after BD to use it as a "fetal container." Such a decision should not be simply assumed, but it must be debated. If the mother is to be considered a "cadaveric incubator" with no autonomous rights, the rights of the fetus should legally prevail. Another argument claims that the prolonged somatic support itself is actually organ donation with the fetus as the recipient. In that case, if the mother had previously indicated a wish to donate her organs, it would be appropriate to proceed with the extended somatic support. Finally, some believe that strategies used to maintain maternal somatic function are still in the experimental stage. Not every adverse effect of medication used on the fetus during an extended somatic support is known. The next of kin must therefore be informed about the existing life maintenance strategies and the possible damages they may cause to the fetus. Psychological consultation should certainly be beneficial in this situation.

Since such catastrophic cases are so infrequent, the mother's wish is in effect rarely known. For this reason, it is strongly suggested to engage the family in the planning of the care. The physician and transplant coordinator should not impose all available procedures against the wishes of the family. Sperling *et al*. [46] suggested that questions be answered on a case-by-case basis with the involvement of the hospital's ethics committee. One also needs to consider that while nowadays somatic support in the case of maternal BD is technically possible, there is still no legal document which asks a pregnant woman about the fate of her unborn child in the event of BD. It is highly recommended that this question be added to the advance directives of any woman of childbearing age and routinely discussed in standard prenatal interviews [47].

## Conclusions

At present, BD is a medically and legally accepted event allowing a pronouncement of death. Taking into account that in maternal BD two organisms are involved, the mother and the fetus, a decision whether to maintain the mother's vital functions to allow fetal survival is also an ethical and legal issue. The goal of prolonged maternal somatic support is to deliver a viable and healthy infant with a beneficial long-term outcome. From the medical point of view, the management of a brain-dead pregnant woman should follow the common standards, guidelines and recommendations for organ-preserving therapy. In some situations, however, the mother needs special medical support and interventions which differ from somatic support in nonpregnant BD patients. Both after a successful delivery and in the case of fetal abortion, the mother can also be considered as an organ donor. In general, we recommend that there be no clear lower limit to the gestational age which would restrict the physician's efforts to support the brain-dead mother and her fetus. A meeting of the neurosurgical, critical care, obstetric, neonatal, transplant and ethical staff, along with the patient's family, should collectively make a decision about future treatment steps. Since currently there are still only a limited number of cases describing the management of extended maternal somatic support after brain death, the current recommendations should be continuously reassessed and adapted along with the growing experience and knowledge. For such serious and rare cases as described here, it would be advisable from a clinical point of view to establish an international registry network of BD pregnant patients, which could help to gather further experience. We also think that from the practical point of view, it would be possible to establish such a registry and this network could become a part of routine clinical usage in all neurosurgery and intensive care centers.

## Competing interests

The authors declare that they have no competing interests.

## Authors' contributions

ME and EK participated in the design of the study and reviewed articles. ME, CD and FSH participated in the design of the study and drafted the manuscript. MG and HF performed the statistical analysis and revised the manuscript. JS, AU, ME, SH and GE were involved in drafting the manuscript or revising it critically for important intellectual content. ME, AM and RA revised the manuscript and gave final approval of the version to be published. All authors read and approved the final manuscript.

## Pre-publication history

The pre-publication history for this paper can be accessed here:

http://www.biomedcentral.com/1741-7015/8/74/prepub

## References

[B1] Mollaret P Goulon M Le coma dépassé Rev Neurol (Paris) 1959 101 3 15 14423403

[B2] Suddaby EC Schaeffer MJ Brigham LE Shaver TR Analysis of organ donors in the peripartum period J Transpl Coord 1998 8 35 39 9726218 10.7182/prtr.1.8.1.c731481782k1uh07

[B3] Slattery MM Morrison JJ Preterm delivery Lancet 2002 360 1489 1497 10.1016/S0140-6736(02)11476-0 12433531

[B4] Wood KE Becker BN McCartney JG D'Alessandro AM Coursin DB Care of the potential organ donor N Engl J Med 2004 351 2730 2739 10.1056/NEJMra013103 15616207

[B5] Holmquist M Chabalewski F Blount T Edwards C McBride V Pietroski R A critical pathway: guiding care for organ donors Crit Care Nurse 1999 19 84 98 10401306

[B6] Shemie SD Ross H Pagliarello J Baker AJ Greig PD Brand T Cockfield S Keshavjee S Nickerson P Rao V Guest C Young K Doig C Pediatric Recommendations Group Organ donor management in Canada: recommendations of the forum on Medical Management to Optimize Donor Organ Potential CMAJ 2006 174 S13 S32 16534070 10.1503/cmaj.045131 PMC1402396

[B7] Drory Y Quaknine G Kosary IZ Kellermann JJ Electrocardiographic findings in brain death: description and presumed mechanism Chest 1975 67 425 432 10.1378/chest.67.4.425 1122770

[B8] Malliani D Peterson DF Bishop VS Brown AM Spinal sympathetic cardio-cardiac reflexes Circ Res 1972 30 158 166 5061316 10.1161/01.res.30.2.158

[B9] Dictus C Vienenkoetter B Esmaeilzadeh M Unterberg A Ahmadi R Critical care management of potential organ donors: our current standard Clin Transplant 2009 23 Suppl 21 2 9 10.1111/j.1399-0012.2009.01102.x 19930309

[B10] Hoemme R Neeser G Organ donation Anesthesist 2007 56 1291 1302 10.1007/s00101-007-1284-8 18080078

[B11] Powner DJ Variables during care of adult donors that can influence outcomes of kidney transplantation Prog Transplant 2005 15 219 224 16252627 10.1177/152692480501500304

[B12] Mallampalli A Guy E Cardiac arrest in pregnancy and somatic support after brain death Crit Care Med 2005 33 325 331 10.1097/01.CCM.0000182788.31961.88 16215355

[B13] Rosengrad BR Feng RA Alfrey EJ Zaroff JG Emond JC Henry ML Garrity ER Roberts JP Wynn JJ Metzger RA Freeman RB Port FK Merion RM Love RB Busuttil RW Delmonico FL Report of the crystal city meeting to maximize the use of organs recovered from the cadaver donor Am J Transplant 2002 2 701 711 10.1034/j.1600-6143.2002.20804.x 12243491

[B14] Field DR Gates EA Creasy RK Jonsen AR Laros RK Jr Maternal brain death during pregnancy JAMA 1988 260 816 822 10.1001/jama.260.6.816 3392814

[B15] Hevesi ZG Angelini G Coursin DB Supportive care after brain death for the donor candidate Int Anesthesiol Clin 2006 44 21 34 10.1097/01.aia.0000210798.53007.4b 16832204

[B16] Feldman DM Borgida AF Rodis JF Campbell WA Irreversible maternal brain injury during pregnancy: a case report and review of the literature Obstet Gynecol Surv 2000 55 708 714 10.1097/00006254-200011000-00023 11075735

[B17] Bhatia P Bhatia K Pregnancy and the lungs Postgrad Med J 2000 76 683 689 10.1136/pmj.76.901.683 11060141 PMC1741787

[B18] Milliez J Cayol V Palliative care with pregnant women Best Pract Res Clin Obstet Gynaecol 2001 15 323 331 10.1053/beog.2000.0172 11358406

[B19] Gramm HJ Meinhold H Bickel U Zimmermann J von Hammerstein B Keller F Dennhardt R Voigt K Acute endocrine failure after brain death? Transplantation 1992 54 851 857 10.1097/00007890-199211000-00016 1332223

[B20] Howlett TA Keogh AM Perry L Touzel R Rees LH Anterior and posterior pituitary function in brain-stem-dead donors: a possible role for hormonal replacement therapy Transplantation 1989 47 828 834 10.1097/00007890-198905000-00016 2718243

[B21] Van Runnard Heimel PJ Franx A Schobben AF Huisjes AJ Derks JB Bruinse HW Corticosteroids, pregnancy, and HELLP syndrome Obstet Gynecol Surv 2005 60 57 70 10.1097/01.ogx.0000150346.42901.07 15618920

[B22] Smith M Physiologic changes during brain stem death: lessons for management of the organ donor J Heart Lung Transplant 2004 23 9 Suppl S217 S222 10.1016/j.healun.2004.06.017 15381167

[B23] Zhu XA Li Y Effect of hyperthermia on the red-cell immune function of rats and its teratogenicity on developing embryos Wei Sheng Yan Jiu 2000 29 89 91 12725083

[B24] Miller MW Nyborg WL Dewey WC Edwards MJ Abramowicz JS Brayman AA Hyperthermic teratogenicity, thermal dose and diagnostic ultrasound during pregnancy: implications of new standards on tissue heating Int J Hyperthermia 2002 18 361 384 10.1080/02656730210146890 12227925

[B25] Mallampalli A Powner DJ Gardner M Cardiopulmonary resuscitation and somatic support of the pregnant patient Crit Care Clin 2004 20 747 761 10.1016/j.ccc.2004.05.005 15388200

[B26] Badgett T Feingold M Total parenteral nutrition in pregnancy: case review and guidelines for calculating requirements J Matern Fetal Med 1997 6 215 217 10.1002/(SICI)1520-6661(199707/08)6:4<215::AID-MFM5>3.0.CO;2-M 9260118

[B27] Dominguez-Roldan JM Murillo-Cabezas F Santamaria-Mifsut JL Munoz-Sanchez A Villen-Nieto J Barrera-Chacon JM Changes in resting energy expenditure after development of brain death Transplant Proc 1995 27 2397 2398 7652852

[B28] Rivera-Alsina ME Saldana LR Stringer CA Fetal growth sustained by parenteral nutrition in pregnancy Obstet Gynecol 1984 64 138 141 6429589

[B29] Hamaoui E Hamaoui M Nutritional assessment and support during pregnancy Gastroenterol Clin North Am 2003 32 59 121 10.1016/S0889-8553(02)00132-2 12635414

[B30] Powner DJ Bernstein IM Extended somatic support for pregnant women after brain death Crit Care Med 2003 31 1241 1249 10.1097/01.CCM.0000059643.45027.96 12682499

[B31] Farragher RA Laffey JG Maternal brain death and somatic support Neurocrit Care 2005 3 99 106 10.1385/NCC:3:2:099 16174876

[B32] Catanzarite VA Willms DC Holdy KE Gardner SE Ludwig DM Cousins LM Brain death during pregnancy: tocolytic therapy and aggressive maternal support on behalf of the fetus Am J Perinatol 1997 14 431 434 10.1055/s-2007-994175 9263566

[B33] Bernstein IM Watson M Simmons GM Catalano PM Davis G Collins R Maternal brain death and prolonged fetal survival Obstet Gynecol 1989 74 434 437 11655965

[B34] Korzeniowski OM Antibacterial agents in pregnancy Infect Dis Clin North Am 1995 9 639 651 7490437

[B35] Einarson A Shuhaiber S Koren G Effects of antibacterials on the unborn child: what is known and how should this influence prescribing Paediatr Drugs 2001 3 803 816 10.2165/00128072-200103110-00003 11735666

[B36] Christensen B Which antibiotics are appropriate for treating bacteriuria in pregnancy? J Antimicrob Chemother 2000 46 Suppl 1 29 34 10.1093/jac/46.suppl_1.29 11051621

[B37] Villa-Forte Gomes MP Venous thromboembolism in pregnancy Curr Treat Options Cardiovasc Med 2009 11 104 113 10.1007/s11936-009-0011-y 19289023

[B38] Lawson EE Antenatal corticosteroids: too much of a good thing? JAMA 2001 286 1628 1630 10.1001/jama.286.13.1628 11585487

[B39] Ferguson S Allen VM Craig C Allen AC Dodds L Timing of indicated delivery after antenatal steroids in preterm pregnancies with severe hypertension Hypertens Pregnancy 2009 28 63 75 10.1080/10641950802366237 19165671

[B40] King JF Flenady VJ Papatsonis DN Dekker GA Carbonne B Calcium channel blockers for inhibiting preterm labour Cochrane Database Syst Rev 2002 2 CD002255 12076443 10.1002/14651858.CD002255

[B41] Haas DM Imperiale TF Kirkpatrick PR Klein RW Zollinger TW Golichowski AM Tocolytic therapy: a meta-analysis and decision analysis Obstet Gynecol 2009 113 585 594 19300321 10.1097/AOG.0b013e318199924a

[B42] Lewis DD Vidovich RR Organ recovery following childbirth by a brain-dead mother: a case report J Transpl Coord 1997 7 103 105 9505652 10.7182/prtr.1.7.3.e47h65u1v846085u

[B43] Lawson EE Antenatal corticosteroids: too much of a good thing? JAMA 2001 286 1628 1630 10.1001/jama.286.13.1628 11585487

[B44] Webb GW Huddleston JF Management of the pregnant women who sustains severe brain damage Clin Perinatol 1996 23 453 464 8884119

[B45] Sheikh AA Cusack DA Maternal brain death, pregnancy and the foetus: the medico-legal implications for Ireland Med Law 2004 23 237 250 15270467

[B46] Sperling D Maternal brain death Am J Law Med 2004 30 453 500 15651556 10.1177/009885880403000402

[B47] Catlin A Volat D When the fetus is alive but the mother is not: critical care somatic support as an accepted model of care in the twenty first century? Crit Care Nurs Clin N Am 2009 21 267 276 10.1016/j.ccell.2009.01.004 19460668

[B48] Dillon WP Lee RV Tronolone MJ Buckwald S Foote RJ Life support and maternal brain death during pregnancy JAMA 1982 248 1089 1091 10.1001/jama.248.9.1089 7109202

[B49] Heikkinen JE Rinne RI Alahuhta SM Lumme JA Koivisto ME Kirkinen PP Sotaniemi KA Nuutinen LS Järvinen PA Life support for 10 weeks with successful fetal outcome after fatal maternal brain damage BMJ 1985 290 1237 1238 10.1136/bmj.290.6477.1237 3921171 PMC1415860

[B50] Antonini C Alleva S Campailla MT Pelosi G Valle E Verrua M Zamponi E Blanda A Gambaro C Morte cerebrale e sopravvivebza fetale prolungata [Brain death and prolonged fetal survival] Minerva Anestesiol 1992 58 1274 1252 1294907

[B51] Nettina M Santos E Ascioti KJ Barber MA Sheila's death created many rings of life Nursing 1993 23 44 48 8446316

[B52] Anstotz C Should a brain-dead pregnant woman carry her child to full term? The case of the "Erlanger baby." Bioethics 1993 7 340 350 10.1111/j.1467-8519.1993.tb00224.x 11651609

[B53] Béguin F Intruduction: La mort cerebrale maternelle [Introduction: maternal cerebral brain death] Arch Gynecol Obstet 1993 253 Suppl S1 S3 10.1007/BF02346790 8117154

[B54] Wuermeling HB Brain-death and pregnancy Forensic Sci Int 1994 69 243 245 10.1016/0379-0738(94)90387-5 7860009

[B55] Iriye BK Asrat T Adashek JA Carr MH Intraventricular haemorrhage and maternal brain death associated with antepartum cocaine abuse Br J Obstet Gynaecol 1995 102 68 69 7833317 10.1111/j.1471-0528.1995.tb09031.x

[B56] Vives A Carmona F Zabala E Fernandez C Cararach V Iglesias X Maternal brain death during pregnancy Int J Gynaecol Obstet 1996 52 67 69 10.1016/0020-7292(95)02535-9 8620992

[B57] Spike J Brain death, pregnancy and posthumous motherhood J Clin Ethics 1999 10 57 65 10394539

[B58] Beca JP Wells W Rubio R Muerte cerebral materna durante el embarazo Rev Med Chile 1998 126 450 455 9699377

[B59] Lane A Westbrook A Grady D O'Connor R Counihan TJ Marsh B Laffey JG Maternal brain death: medical, ethical and legal issues Intens Care Med 2004 30 1484 1486 10.1007/s00134-004-2305-6 15107974

[B60] Hussein IY Govenden V Grant JM Said MR Prolongation of pregnancy in a woman who sustained brain death at 26 weeks of gestation BJOG 2006 113 120 122 16398782 10.1111/j.1471-0528.2005.00801.x

[B61] Souza JP Oliveira-Neto A Surita FG Cecatti JG Amaral E Pinto e Silva JL The prolongation of somatic support in a pregnant woman with brain-death: a case report Reprod Health 2006 3 3 10.1186/1742-4755-3-3 16643646 PMC1459115

[B62] Mejia R Badariotti G De Diego B Ridruejo O O'Flaherty E Brain death in a pregnant woman and fetus survival Medicina (Buenos Aires) 2008 68 447 452 19147427

